# Genomic and Functional Studies of *Drosophila* Sex Hierarchy Regulated Gene Expression in Adult Head and Nervous System Tissues

**DOI:** 10.1371/journal.pgen.0030216

**Published:** 2007-11-23

**Authors:** Thomas D Goldman, Michelle N Arbeitman

**Affiliations:** 1 Section of Molecular and Computational Biology, Department of Biological Sciences, University of Southern California Los Angeles, Los Angeles, California, United States of America; 2 Section of Neurobiology, Department of Biological Sciences, University of Southern California Los Angeles, Los Angeles, California, United States of America; Stanford University Medical Center, United States of America

## Abstract

The *Drosophila* sex determination hierarchy controls all aspects of somatic sexual differentiation, including sex-specific differences in adult morphology and behavior. To gain insight into the molecular-genetic specification of reproductive behaviors and physiology, we identified genes expressed in the adult head and central nervous system that are regulated downstream of sex-specific transcription factors encoded by *doublesex* (*dsx*) and *fruitless* (*fru*). We used a microarray approach and identified 54 genes regulated downstream of *dsx*. Furthermore, based on these expression studies we identified new modes of DSX-regulated gene expression. We also identified 90 and 26 genes regulated in the adult head and central nervous system tissues, respectively, downstream of the sex-specific transcription factors encoded by *fru*. In addition, we present molecular-genetic analyses of two genes identified in our studies, *calphotin* (*cpn*) and *defective proboscis extension response* (*dpr*), and begin to describe their functional roles in male behaviors. We show that *dpr* and *dpr*-expressing cells are required for the proper timing of male courtship behaviors.

## Author Summary

The fruit fly *Drosophila* is an excellent model system to use to understand the molecular-genetic basis of male courtship behavior, as the potential for this behavior is specified by a well-understood genetic regulatory hierarchy, called the sex determination hierarchy. The sex hierarchy consists of a pre-mRNA splicing cascade that culminates in the production of sex-specific transcription factors, encoded by *doublesex (dsx)* and *fruitless (fru)*. *dsx* specifies all the anatomical differences between the sexes, and *fru* is required for all aspects of male courtship behavior. In this study, we measure gene expression differences between males and females, and between sex hierarchy mutants and wild-type animals, to identify genes that underlie the differences between males and females. We have performed these studies on adult head and nervous system tissues, as these tissues are important for establishing the potential for behaviors. We have identified several genes regulated downstream of *dsx* and *fru* and more extensively characterized two genes that are more highly expressed in males. One gene regulated downstream of *dsx* is expressed in the retina and is known to have a function in visual transduction. The other gene, regulated downstream of *fru*, plays a role in the timing of male courtship behavior.

## Introduction

Genomic microarray approaches have allowed scientists to address questions that were previously intractable using molecular-genetic approaches. For example, in studies of *Drosophila*, microarrays can facilitate the identification of genes that underlie complex behaviors in the adult. These genes are difficult to identify using standard genetic approaches, as most genes are utilized during early development [1]. Given the likelihood of a developmental phenotype, adult-specific function is difficult to ascertain. The terminal genes in the *Drosophila* sex hierarchy encode sex-specific transcription factors that have been shown to play key roles in specifying sex-specific behaviors. Animals with mutations in the genes that encode these transcription factors, and that display adult-specific behavioral phenotypes, are particularly well suited for the identification of genes that underlie complex behaviors. In this study, we use genomic approaches to identify genes that are regulated downstream of the sex hierarchy transcription factors, thereby providing insight into the molecular basis of male courtship behavior.

**Figure 1:**
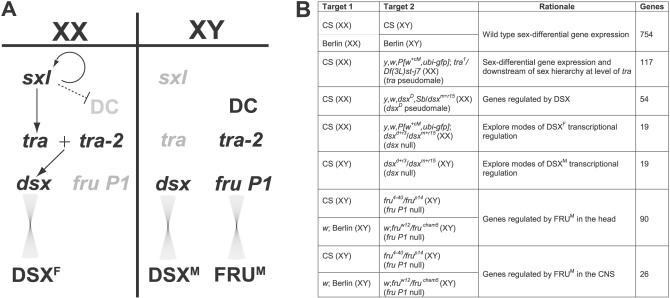
The Sex Determination Hierarchy and Microarray Experimental Overview (A) The *Drosophila* sex determination hierarchy consists of an alternative pre-mRNA splicing cascade, culminating in the production of sex-specific transcription factors encoded by *dsx* and *fru P1*. In *Drosophila*, the primary determinant of sex is the X chromosome to autosome ratio. In females, this ratio is one and leads to the production of SXL. *sxl*, *tra*, and *tra-2* encode pre-mRNA splicing factors. SXL regulates *sxl* and *tra* pre-mRNA splicing, resulting in the production of TRA in females. TRA together with TRA-2 regulates the splicing of *dsx* and *fru P1* pre-mRNAs. In males, *dsx* and *fru P1* are spliced by the default pathway. Arrows indicate regulation of pre-mRNA splicing, and hourglass shapes indicate translation. The dotted line indicates inhibition of the dosage compensation (DC) pathway. (B) Experimental overview for microarray comparisons. The genotypes and phenotypes of animals from which the microarray probe pairs are derived are indicated in columns with headings Target 1 and Target 2. For each genotype, the chromosomal sex is shown in parentheses. The rationale for the experimental comparison is indicated. The number of genes that met the statistical criteria (see text) is indicated in the column with heading “Genes.” doi:0.1371/journal.pgen.0030216.g001

The *Drosophila* sex determination hierarchy consists of an alternative pre-mRNA splicing cascade that culminates in the production of sex-specific transcription factors encoded by *doublesex* (*dsx*) and *fruitless* (*fru*) (Figure 1) (reviewed in [2]). *dsx* produces both male- and female-specific transcription factors (DSX^M^ and DSX^F^, respectively) [3]. *fru* is a complex locus with at least four promoters; it is the product of the *P1* promoter (*fru P1*) that is sex specifically spliced and produces male-specific FRU isoforms (FRU^M^) [4].

In *Drosophila*, male courtship behavior is an innate, genetically programmed behavior that consists of a series of steps performed by the male to attract a mate (reviewed in [5]). The male orients towards the female, follows her, taps her with his foreleg, produces a species-specific courtship song via wing vibration, contacts the female genitalia with his proboscis, and then attempts copulation. If the female has not recently mated, she typically will allow copulation to proceed. *fru P1* is necessary for specifying the potential for all of these male courtship steps [4,6–9]. In addition, when FRU^M^ is produced in females, in the homologously positioned neurons in which it is normally produced in males, the early steps of the male courtship ritual are observed [10,11]. *dsx* is required for specifying all adult morphological differences between the sexes [12], most of which are required for courtship performance [13,14]. *dsx* also functions in the central nervous system (CNS) to specify the potential for courtship wing song and wild-type levels of courtship performance [13–17].

The genes regulated downstream of *dsx* and *fru P1* remain largely unknown in most tissues of the adult fly. Several genes have been identified that are regulated downstream of DSX activity in the adult internal genitalia [18], and the Yolk protein genes have been shown to be direct targets of DSX in fat-body tissues [19–21]. Genes shown to be regulated downstream of FRU^M^ activity, include *yellow* [22], *takeout* [23], and *neuropeptide F* [24].

To gain insight into how sex-specific behaviors are specified, sex-differential gene expression was examined in adult head tissue and dissected CNS tissue. Using a microarray approach, we have identified genes regulated by the sex hierarchy that are either direct or indirect targets of DSX and/or FRU^M^, in either adult head tissues or in the CNS. By extending the gene expression analyses, we have also determined new modes of DSX-regulated gene expression. We present additional molecular-genetic analyses of two genes, *calphotin* (*cpn*) and *defective proboscis extension response* (*dpr*). We demonstrate that *cpn* is more highly expressed in the retina in males, as compared to females, and is downstream of DSX activity. In addition, we show that *dpr* is regulated downstream of *fru P1* and is expressed in *fru P1*-expressing cells in the CNS. We demonstrate a role for *dpr* and for *fru P1* in *dpr*-expressing cells in male courtship performance.

## Results/Discussion

### Overview of Approach to Determine Regulation of Sex-Differentially Expressed Genes in Adult Head Tissues

A major goal of this work is to understand, at a molecular-genetic level, how sex-differential gene expression is established in adult head tissues, to gain insight into how the potential for reproductive behaviors are established. Here, we analyzed gene expression using a glass-slide microarray approach (see Materials and Methods). For each sex-differentially expressed gene identified, we sought to determine at which level or branch of the somatic sex hierarchy, sex-differential expression is established. This will provide an understanding of how a multitiered and branched genetic regulatory hierarchy deploys the genome to bring about developmental and physiological differences. The logic of our approach is as follows (see Figure 1B). We first compared gene expression between wild-type male and female adult heads to identify the genes that are differentially expressed between the sexes. We next determined if regulation of the sex-differentially expressed genes is downstream of *transformer* (*tra)* (see Figure 1). A gene that is sex differentially expressed and not regulated downstream of *tra* would be inferred to be regulated at the level of *sxl*, or because of differences in sex chromosome composition (Figure 1A). *tra* and *sxl* both encode pre-mRNA splicing factors. *sxl* is at the top of both the *tra* branch and the branch of the sex hierarchy that controls dosage compensation, the process that equalizes the amount of transcript produced from the single male X chromosome to that of the two female X chromosomes (Figure 1A; reviewed in [2,25]). For those genes that are regulated downstream of *tra*, we distinguished between possible regulation by sex-specific transcription factors encoded by *dsx* and *fru P1* by performing additional gene expression analyses. Finally, we distinguished between sex-differential expression in the CNS versus other tissues in the adult head.

### Sex-Differentially Expressed Genes in the Adult Head

We first identified genes that are sex differentially expressed in wild-type adult head tissues. We compared gene expression in 0–24-h adult male and female heads, from two different wild-type strains, Canton Special (CS) and Berlin. We used two strains to ensure that we focused on key genes underlying the differences between the sexes. To identify genes with significant differences in gene expression, a False Discovery Rate (FDR) method was employed [26]. FDR is the proportion of false positives among all the genes initially declared as being differentially expressed. FDR has become a standard for multiple testing paradigms such as whole-genome microarray analyses [27,28]. Throughout this study, we used an FDR cutoff of 0.15 (*q* < 0.15), unless otherwise noted. In our analyses, candidate genes passed the FDR cutoff for multiple independent array experiments. Thus, we reasoned that allowing up to 15% of all declared differentially expressed genes within each test as false positives would be a conservative cutoff to obtain high confidence in our analyses.

We combined the data from the two wild-type strains for statistical analyses, with the expectation that sex-differentially expressed genes would show the same direction of change in the two strains. We identified 754 genes that displayed significant, sex-differential expression (see Materials and Methods). For these 754 genes, the range of expression values was between ∼130-fold higher in females to ∼45-fold higher in males, with 94% of genes displaying differences less than 2-fold. Three hundred thirty and 424 genes were more highly expressed in males and females, respectively. Of these 754 genes, 46 genes displayed both significant and substantial sex-differential expression (*q* < 0.15 and fold change [FC] > 2; Table S1).

We confirmed our array experiments accurately identified sex-differentially expressed genes. First, we identified several genes previously shown to display robust sex-differential expression. In particular, *Yolk protein 1* (*Yp1*), *Yolk protein 2* (*Yp2*), and *Yolk protein 3* (*Yp3*) are highly expressed in the female, but not male, adult fat body [29,30]. Consistent with this, we observed over 100 times more *Yp1* and *Yp2* transcripts, and 40 times more *Yp3* transcript in adult head tissues from females, than males. Second, we analyzed data from array features that are specific for the transcripts *CG11094-RA* and *CG11094-RB* that produce DSX^M^ and DSX^F^ isoforms, respectively. *CG11094-RA* and *CG11094-RB* showed significantly higher expression in wild-type males and females, respectively. Finally, several genes in this list have previously been shown to display sex-differential expression, such as *female-specific independent of transformer* (*fit*) and female-enriched *sex-specific enzyme 2* (*sxe2*) [31].

### Sex-Differentially Expressed Genes in Adult CNS Tissues

To distinguish between sex differences in gene expression in the CNS from those contributed by other tissues of the head, such as the fat body, we compared gene expression between male and female dissected brain and ventral nerve cord tissues. We identified four genes, including *fit* and *CG8007*, a male-biased gene inferred to be involved in serotonin receptor signaling [32], with significant, sex-differential expression. The other two genes are *CR32998* and *rna on the x2* (*rox2,* CR32665), both of which encode small RNAs and are more highly expressed in males. *CR32998* encodes a transcript with sequence similarity to small nuclear RNAs (*snRNA, U425F*), and *rox2* encodes a RNA component of the dosage compensation complex. In *Drosophila*, the dosage compensation complex mediates up-regulation of gene expression on the X chromosome in males. *rox2* RNA is part of the dosage compensation complex in males [33–35], further validating our microarray data.

The identification of only four genes with sex-differential expression in the CNS is not due to poor quality data, as we had the power to detect 94.7% of gene expression differences (see Materials and Methods). The Pearson correlation statistic among all hybridizations for these experiments was *r^2^* = 0.96 (see Materials and Methods), further demonstrating low experimental variation among our replicates. Thus, most sex-differential expression in adult head tissue we detected was because of expression outside of the CNS. Our findings are consistent with previous studies that have suggested that there are hundreds of genes expressed in the adult head fat body that are not expressed in the CNS [36]. It is possible that many fat body–specific mRNAs are sex differentially expressed. Indeed, previous studies have identified sex-biased fat body–specific mRNAs, such as *Cyp4d21* [23,36], *turn on sex-specificity* (*tsx*), *sxe2* [31], *drosomycin* [37], and mRNAs from the gene family *takeout* [23,36,38].

In our microarray data analyses of dissected CNS tissue, *fit* showed significantly higher expression in females than males (see above), similar to a previous study that detected female-enriched expression of *fit*, largely in head fat cells [31]. Even if *fit* were solely expressed in the fat body, this is the only gene previously identified as expressed in the fat body that was identified in our analyses of gene expression in the CNS, suggesting that our dissections minimized contamination from peripheral perineuronal fat-body tissues. Our data indicate that many genes are expressed at similar levels in males and females, when gene expression in the entire CNS is assayed. However, these experiments would not allow for the identification of genes that display sex-specific spatial expression differences, but that overall are expressed at similar levels in males and females, or genes that are sex differentially expressed, but below the limit of our microarray detection, such as those genes that are expressed in very few cells of the CNS. Indeed, we did not detect *dsx*, *yellow*, *or npf*, in these CNS microarray comparisons, though we know that they are sex differentially expressed in the CNS [15,24,39,40].

### Sex-Differentially Expressed Genes in Adult Head Tissue Regulated Downstream of *tra*

**Figure 2:**
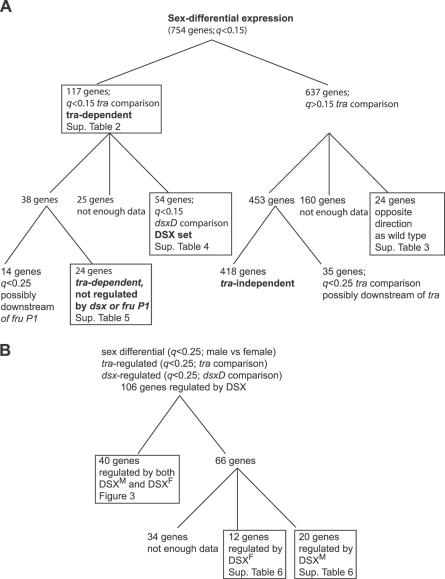
Overview of Results from Statistical Tests Performed on Microarray Data (A) Overview of results from statistical tests of microarray data leading to the identification of sex-differentially expressed genes regulated by *tra*, *dsx*, or neither in adult head tissues. (B) Overview of results from statistical tests of microarray data leading to the identification of genes regulated downstream of DSX activity and the modes of DSX regulation for these genes. doi:0.1371/journal.pgen.0030216.g002

We next sought to identify genes that are regulated downstream of *tra* in adult head tissues (Figure 1A). We compared gene expression in *tra* mutant animals (*tra* pseudomales) to wild-type females. The *tra* pseudomales are chromosomally XX, but are phenotypically almost identical to wild-type males. *tra* is required for splicing *dsx* and *fru P1* pre-mRNAs in females. Thus, *tra* pseudomales produce both DSX^M^ and FRU^M^, which direct male somatic development (Figure 1A). We identified 117 genes that are sex differentially expressed between wild-type males and females, and between *tra* pseudomales and wild-type females (one-tailed *t*-test) (Table S2); 32 and 85 genes are more highly expressed in males and females, respectively. The remaining 637 genes out of the 754 sex-differentially expressed genes either (1) showed no significant differential expression (613 genes), or (2) showed significant differential expression in the opposite direction than in wild-type males versus females (24 genes, see Figure 2A).

### Sex-Differential Gene Expression That Is Independent of *tra*

We examined the data for the 613 genes that showed no significant difference in expression in the *tra* comparison, to further determine if the sex-differential expression we observed in wild-type animals is independent of *tra*. One hundred sixty out of 613 genes were missing data in one or more of the four experimental replicates. For these 160 genes we had much less statistical power, compared to what we had in our experiments analyzing wild-type animals (eight replicates) and so were not further considered. We next examined the data for the remaining 453/613 genes, for which we had data in all four of the *tra* comparisons. If we reduced the stringency of our statistical cut-off (*q <* 0.25), 35/453 showed significant differential expression downstream of *tra* and so might be regulated downstream of TRA activity (see Figure 2A).

This suggests that the remaining 418/453 genes are sex differentially expressed, but are not downstream of *tra*. Indeed, as expected for a gene regulated independent of *tra*, nearly all of the 418 genes are also not downstream of *dsx* and/or *fru P1*. Only 19/418 genes were significantly differentially expressed in our *dsx* and/or *fru P1* microarray comparisons and thus are possible false negatives in the *tra* data analyses (see below and Figure 1A).

For the 418 genes, the median and average fold difference in expression between males and females is 1.14 and 1.07, respectively. One possibility is that for these 418 genes, the small fold differences in gene expression between the sexes are due to having different numbers of sex chromosomes between wild-type males and females, and/or the efficiency of dosage compensation, which is regulated at the level of *sxl*. In the sex hierarchy, *sxl* is above the level of *tra* (see Figure 1A). In the *tra* pseudomale to female comparison, *tra* pseudomales and females are both chromosomally XX and produce SXL, so gene expression differences due to differences in sex chromosomes and/or dosage compensation would be eliminated. To determine if the 418 genes we identified as sex differentially expressed, but not dependent on *tra*, are due to differences in sex chromosome composition, we determined the chromosomal distribution of the 418 genes. We observed a significant over-representation of genes on the X chromosome (*p =* 2.25E-07, hypergeometric test), but no significant over-representation of genes on any other chromosome. Taken together, these observations suggest that these 418 genes that display small fold differences in sex-differential gene expression and are not regulated downstream of *tra*. It appears they are regulated at the level of *sxl* and dosage compensation and/or because of differences in the number of sex chromosomes between males and females.

Of the 24 genes for which expression was in the opposite direction from wild-type males versus females, six were more highly expressed in females compared to *tra* pseudomales, and 18 were more highly expressed in *tra* pseudomales than wild-type females (Table S3). The expression profiles of these 24 genes may reflect complex modes of sex-hierarchy regulation, as previously described in pupal-distal-leg segments [41]. However, given the fact that for these 24 genes there is significant over-representation (*p* < 6.6E-3) of genes involved in stress-induced humeral defense against bacterial infection or high temperature, including *turandot A*, *turandot C* [42], and *immune induced molecule* [43], this suggests that many of these 24 genes are not sex-hierarchy regulated, but are induced by infection or stress.

### Sex-Differentially Expressed Genes in Adult Head Tissue Regulated Downstream of *dsx*

Next, we identified genes that are regulated by DSX activity in adult heads. Here we compared gene expression in chromosomally XX, *dsx^D^* pseudomales to wild-type females. The *dsx^D^* pseudomales are transheterozygous for one *dsx* null allele and one *dsx* allele that can only produce DSX^M^. These animals look almost identical to wild-type males, as DSX^M^ directs male somatic development and physiology. We employed one-tailed statistical tests, reasoning that we could predict the direction of gene expression in *dsx^D^* pseudomales comparisons, based on the wild-type and *tra* pseudomale array comparisons (Table S2). We identified 54 genes that displayed significant sex-differential expression between wild-type males and females, as well as significant sex-differential expression between *tra* pseudomales and females, and *dsx^D^* pseudomales and females (one-tailed *t*-tests; Table S4). Forty-seven and seven genes were more highly expressed in females and males, respectively. These 54 genes will hereafter be referred to as the “DSX set.”

We identified functional annotation categories that are enriched in the DSX set, to infer processes regulated by DSX. The functional annotation analysis tool Database for Annotation, Visualization, and Integrated Discovery (DAVID) can be used to determine significant enrichment of functional annotations of genes in an input gene list, against a background gene list (see Material and Methods) [44]. Using DAVID, we identified metal ion binding (involved in apoptosis) and calcium ion binding (involved in cell signaling) as enriched functional categories in the DSX set (Table S4; *p <* 0.05). DSX might establish sex-specific differences at the morphological level by controlling cell proliferation, perhaps by regulating apoptosis or cell division, as shown previously [45]. DSX effectors may also be involved in controlling cell differentiation and physiology through calcium-mediated cell signaling. In addition, several genes identified in the DSX set have been shown to be involved in light and olfactory sensory responses. Differences in these sensory responses may underlie differences in behavioral outputs of adult males and females. DSX regulation of these genes suggests that DSX has a broader role than just specifying morphological differences between the sexes, but may also play a role in establishing the potential for behavior, in collaboration with FRU^M^.

### Genes Regulated Downstream of *tra* That Are Not Regulated Downstream of *dsx* or *fru P1* in Adult Head Tissues

We identified 117 genes that are sex differentially expressed and regulated downstream of *tra*, but only 54/117 genes are also regulated downstream of DSX activity, leaving open the possibility that the other 63 genes are regulated by *fru P1* or by another branch of the somatic sex hierarchy (see Figures 1A and 2A). Of these 63 genes, we removed 25 genes from consideration, as we either did not have data in all four *dsx^D^* comparisons, or the gene displayed significant differential expression when we employed a less stringent statistical criteria (*q <* 0.25), leaving open the possibility of regulation downstream of *dsx*. Of the 38 remaining genes, 14 genes displayed significant differential expression in the *fru P1* comparisons, suggesting regulation by FRU^M^ (*q <* 0.25, see Materials and Methods and below). Thus, there were 24 genes that displayed significant sex-differential expression downstream of *tra* and were not regulated downstream of *dsx* or *fru P1* (Table S5). This raises the possibility that there is another branch of the sex hierarchy that is responsible for differences in transcript abundances that is at the same level as *dsx* and *fru*. Another transcription factor, *dissatisfaction* (*dsf*) has been postulated to function in the sex hierarchy at the same level as *dsx* and *fru* [46]. Alternatively, it is possible that sex-specific differences in transcript abundance for this set of genes are regulated directly by TRA. In the latter case, a pre-mRNA transcript that is differentially spliced by TRA might have different stability, as compared to one spliced by the default-splicing pathway.

### Modes of DSX Regulation

*Yp1* and *Yp2* are direct targets of DSX that are highly expressed in female fat body tissues and show little or no expression in males [19–21,30]. On the basis of analyses of the *Yp1* and *Yp2* regulatory region, a model for how DSX regulates gene expression has been put forth [21,30]. In this model, the sex-specific DSX isoforms, given their identical DNA binding domains but different *trans*-activation and protein-protein interaction regions, have opposite regulatory functions for a target gene in the two sexes [47,48]. The model is based on the observation that DSX^F^ activates *Yp1* expression in females, and DSX^M^ represses *Yp1* in males. An extension of the model postulated that a male-specific gene would be transcriptionally activated by DSX^M^ activity in males and repressed by DSX^F^ activity in females [21,48–50]. Activation of gene expression by DSX^M^ activity in males, and repression by DSX^F^ activity in females, has been reported for several genes including *takeout* [18,23].

To examine if the model based on *Yp* transcriptional regulation is a general mechanism for how DSX regulates target gene expression, we performed microarray experiments that compared gene expression in adult head tissues of chromosomally XX and XY *dsx* null intersexual animals (*dsx^d+r3^*/*dsx^m+r15^)* to wild-type females and males, respectively. Thus, for a gene that showed sex-differential expression, we could determine if DSX activated, repressed, or had no effect on the expression of the gene, in both males and females. Of the 54 genes in our DSX set, there were 19 genes that showed significant differential expression downstream of *dsx*, in both *dsx* null comparisons (Figure 1B).

The direction of gene expression changes suggests there are at least three different modes of DSX regulation for these 19 genes. Surprisingly, only four of these 19 genes (*Yp1, Yp2*, *Yp3,* and *CG7607*) showed the previously postulated *Yp*-like DSX regulation. Whereas, seven genes showed lower expression in both sexes when there was no DSX. This suggests that these genes are usually activated downstream of *dsx* in both sexes. In contrast, there were eight genes that showed higher expression in both *dsx* null genotypes compared to wild type, suggesting these genes are usually repressed downstream of *dsx* in both sexes. This was similar to what was first observed in our analyses of DSX-regulated gene expression at pupal stages (L. Sanders, M. Lebo, and M. N. Arbeitman, unpublished data).

**Figure 3:**
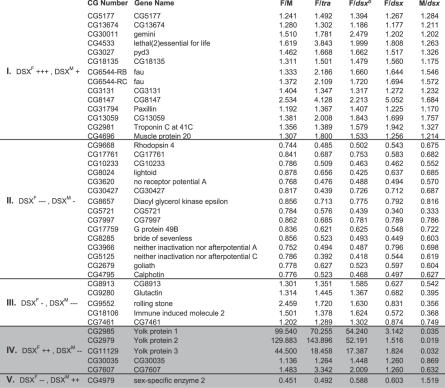
DSX Modes of Regulation The 40 genes listed here showed significant differential expression in wild-type female versus male, female versus *tra* pseudomale, female versus *dsx^D^* pseudomale, female versus *dsx* null XX, and male versus *dsx* null XY in 0–24-h adult heads. Columns contain FC ratio values from the microarray data: F/M, female over male; F/*tra*, female over *tra*; F/*dsx^D^*, female over *dsx^D^*; F/*dsx*, female over *dsx* null XX; M/*dsx*, male over *dsx* null XY. The white region contains genes (34) that showed activation or repression by both DSX^F^ and DSX^M^ in the same direction, in both sexes. The gray region contains genes (six) that showed DSX^F^ and DSX^M^ acting in opposite directions. (I.) Activation by both DSX^F^ and DSX^M^ with higher level of activation by DSX^F^. (II.) Repression by both DSX^F^ and DSX^M^ with higher level of repression by DSX^F^. (III.) Repression by both DSX^F^ and DSX^M^ with higher level of repression by DSX^M^. (IV.) Activation by DSX^F^ and repression by DSX^M^. (V.) Repression by DSX^F^ and activation by DSX^M^. The symbols (+/−) in the first column are qualitative representations of activation (+) or repression (−), based on the microarray data. doi:0.1371/journal.pgen.0030216.g003

Given this unexpected observation, we wanted to have a larger number of genes to examine the modes of DSX regulation. We defined an additional DSX-regulated set using less stringent FDR tests than we previously employed. We identified 106 genes that displayed significant sex-differential expression between both wild-type males and females (*q <* 0.25), and between females versus both *tra* pseudomales and *dsx^D^* pseudomales (one-tailed *t*-tests, *q <* 0.25) (see Figure 2B). Of these 106 genes, 40 genes (Figure 3) also showed significant differential expression in both *dsx* null comparisons, suggesting DSX regulates their expression in both sexes.

We first examined these 40 genes to ascertain if any appeared to be regulated in a manner similar to *Yp1* and identified only six genes (Figure 3, gray region). Five of the six have data consistent with being activated by DSX^F^ in females and repressed by DSX^M^ in males (Figure 3, section IV). We identified one gene, *CG4979* (encodes a predicted sex-specific enzyme involved in lipid metabolism)*,* which appears to be activated by DSX^M^ activity in males and repressed by DSX^F^ activity in females, suggesting that DSX^M^ can be an activator in males, as predicted by the early model and in other studies [18,21,48–52] (Figure 3, section V). Thus, for a small set of genes, DSX regulation of gene expression can be described by the early model that was based on *Yp1* regulation. However, for the genes in this study, regulation by DSX might be direct or indirect.

The majority of genes (34/40 genes) that displayed sex-differential expression downstream of *dsx* in both sexes appear to be activated or repressed downstream of DSX activity in both sexes, but the extent of activation or repression is sex-specific (Figure 3, white region). Of these 34 genes, 14 genes showed significantly lower expression in both chromosomally XX and XY *dsx* null animals, compared to wild-type female and male animals, respectively (Figure 3, section I). 20/34 genes showed significantly higher expression in both chromosomally XX and XY *dsx* null animals, compared to wild-type female and male animals, respectively (Figure 3, sections II and III).

The majority of male-biased genes were repressed by DSX activity in both sexes, but DSX^F^ activity repressed to a greater extent in females than DSX^M^ activity did in males, resulting in higher expression in males. In contrast, the majority of female-biased genes were activated by DSX activity in both sexes, but DSX^F^ activity activated these genes in females to a greater extent than DSX^M^ did in males, resulting in higher expression in females. If any of these genes are direct targets of DSX, this observation is consistent with the fact that the sex-specific region of DSX^F^ interacts with another protein encoded by *intersex*, and this interaction is required for DSX^F^ transcriptional activity, potentially making DSX^F^ a more potent transcriptional activator and repressor than DSX^M^ [53].

We have performed additional microarray experiments in which we individually overexpressed each DSX isoform in adult head tissues. This expression data validated that many genes that appear to be activated or repressed by both DSX isoforms, on the basis of the *dsx* null comparisons, show the predicted expression changes in the ectopic expression experiments (T. D. Goldman and M. N. Arbeitman, unpublished data).

### Genes Regulated by DSX in Only One Sex

Thus far, we have described the modes of DSX regulation for genes that are regulated by *dsx* in both sexes. However, it is possible that DSX activity is only required in one sex for sex-differential expression. This has previously been suggested for four genes expressed in the male accessory gland and one gene expressed in the female spermathecae [18], as well as by others [51,52].

We determined if the remaining 66/106 genes of DSX-regulated genes, identified above, are regulated by DSX in only one sex (see Figure 2B). For 34/66 genes we either did not have sufficient array data, or the data were highly variable and at the borderline of our significance cut-offs above, and so we did not consider these genes further. Twelve of the remaining 32 genes appear to be regulated by DSX^F^ and not DSX^M^. Of these 12 genes regulated only by DSX^F^, nine appear to be activated and three repressed (Table S6). The remaining 20 genes showed significant expression differences in the male *dsx* null comparisons only, suggesting that these genes are regulated only by DSX^M^ and not DSX^F^; here 17 and three genes appeared to be repressed or activated by DSX^M^, respectively. Again, this suggests that the primary mode of DSX^M^ regulation is repression.

Taken together, we have shown that for 106 genes regulated downstream of DSX activity, there are four main modes of regulation: (1) DSX is either an activator or repressor in both sexes, (2) DSX acts as an activator in one sex and a repressor in the other, (3) genes are only regulated by DSX^F^ activity, and (4) genes are only regulated by DSX^M^ activity.

### *calphotin* Is Transcriptionally Regulated by DSX in Adult Head Tissues

We chose one gene, *calphotin* (*cpn*), regulated by DSX to analyze more extensively. CPN is present in photoreceptor cells of the developing eye imaginal disc [54] and functions in rhabdomere and photoreceptor development, as *cpn* mutants display photoreceptor cell death and have misoriented and disrupted rhabdomere structures [55]. In the adult, *cpn* flies lack pigment in some parts of the eye and show a rough eye phenotype [55]. Additionally, adult *cpn* flies show a slight reduction in phototaxis towards visible light [55]. Although rhabdomere structure is abnormal, phototaxis functionality appears to be largely intact, leaving the role of *cpn* in adult physiology an unanswered question. CPN contains a leucine zipper and has been suggested to have calcium-binding properties and play a role in signal transduction by regulating free calcium levels in photoreceptor cells [54,56]. Several genes that are thought to be involved in phototransduction or in light-induced release of internally sequestered calcium ions, including *cpn*, were identified as regulated downstream of DSX activity. This includes *no receptor potential A* (CG3620), *lightoid* (CG8024), *neither inactivation nor afterpotential A* (CG3966), *neither inactivation nor afterpotential C* (CG5125), *G protein 49B* (CG17759), and *bride of sevenless* (CG8285) (see Figure 3).

Given that several genes involved in phototransduction and genes encoding rhodopsins have previously been identified with soma-biased, sex-differential expression in adult flies [37], we wanted to determine if *cpn* might be involved in sex-specific photoreceptor electrophysiological responses in the adult. These sex-specific differences in visual transduction could account for differences in male and female behaviors that require vision, such as differences in circadian activity [57–59] and reproductive behaviors [60–62]. Consistent with the idea that *cpn* may play a role in behaviors, a previous whole-genome array study to identify *clock*-controlled genes identified *cpn* as a gene under circadian regulation in the adult head [59].

**Figure 4:**
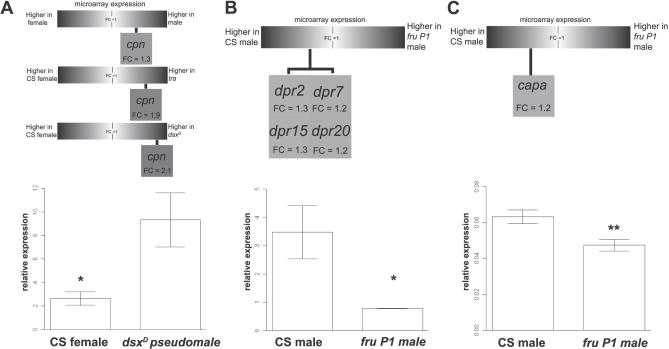
Independent Validation of Microarray Expression Results using RT-PCR Fold-change (FC) from averaged microarray data for each validated gene is shown at the top of each panel. Each genotype of the target pair compared in the microarray experiment is indicated at either side of the gradient bar. White in the gradient bar represents no difference in gene expression between the two genotypes (FC = 1), and the white to black gradient indicates increasingly higher expression differences in the genotypes indicated. The FC in gene expression between the two genotypes is shown below the gradient bar in the gray-scaled box. At the bottom of each panel are the results of RT-PCR analysis, for which the expression of a given gene relative to *rp49* ribosomal expression is reported. Microarray and RT-PCR results for (A) *cpn*, (B) *dpr*-family members, and (C) *capa*. (A) RT-PCR showed that *cpn* expression relative to *rp49* expression was significantly higher (*p* < 0.015, 3.5-fold) in *dsx^D^* female (mean = 9.32, SE = 1.99) than in CS female (mean = 2.64, SE = 0.49) adult heads. (B) *dpr2*, *dpr7*, *dpr14*, and *dpr20* microarray expression, with higher expression in males than *fru P1* males. *dpr* RT-PCR expression relative to *rp49* expression was significantly higher (*p* < 0.05, 4.4-fold) in CS male (mean = 3.47, SE = 0.94) than *fru P1* male (mean = 0.78, SE = 0.004) adult heads. (C) RT-PCR showed that *capa* expression relative to *rp49* expression was significantly higher (*p* < 9.2E-4, 1.4-fold) in CS male (mean = 0.063, SE = 0.003) than *fru P1* male-dissected (mean = 0.047, SE = 0.003) CNS tissue. Error bars represent SE. One-tailed *t*-test: *, *p* < 0.05; **, *p* < 0.001. doi:0.1371/journal.pgen.0030216.g004

We first performed real-time (RT) PCR assays to confirm our microarray results and observed significantly higher *cpn* expression in *dsx^D^* pseudomales than wild-type females (∼3.5-fold, *p <* 0.015; Figure 4A). Our RT-PCR results are consistent with our microarray results where ∼2.2-fold higher *cpn* expression was observed in *dsx^D^* pseudomales than females. On the basis of our *dsx* null microarray comparisons, *cpn* appears to be repressed by DSX in both males and females, but to a greater extent by DSX^F^ than DSX^M^, which ultimately leads to higher expression in males than females.

**Figure 5:**
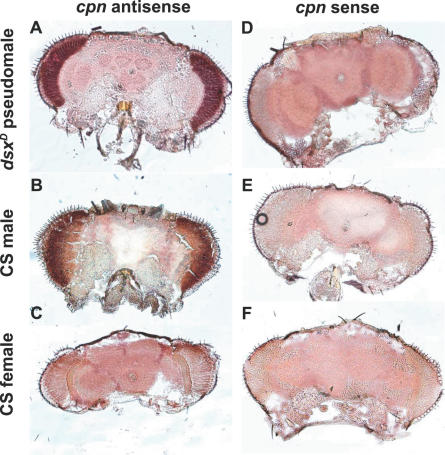
*cpn* Shows Higher Expression in Males and *dsx^D^* Pseudomales than Females and Is Localized to the Retinal Region In all panels, 0–24-h adult head frozen-sections are shown. (A–C) In situ hybridization using *cpn* anti-sense probe. (D–F) In situ hybridization using *cpn* sense control probe. The genotype of the tissue is shown at the left of the panels. doi:0.1371/journal.pgen.0030216.g005

We wanted to determine if sex-differential expression within head tissues was due to *cpn* being expressed in the same regions, but more highly in males than females, or if *cpn* is expressed in broader spatial domains in males than females. Accordingly, we performed frozen section in situ analysis and observed higher expression in *dsx^D^* pseudomales and wild-type males as compared to wild-type females (Figure 5). The observed *cpn* expression is consistent with previous reports of expression restricted to the photoreceptor cells throughout the retina [54,56]. The spatial expression regions are similar in males, females, and pseudomales. This suggests that the sex-specific differences in *cpn* expression levels are not due to broader spatial expression in males, but are due to higher expression in the same tissue in which *cpn* is expressed in females. These results, together with the observation that several genes that underlie visual transduction are sex differentially expressed, suggest that quantitative differences in gene expression in photoreceptor cells between males and females may account for differences in sex-specific visual physiological and behavioral responses.

### Genome-Wide Identification of Genes Regulated by FRU^M^ in Adult Head Tissues

Given the observation that most genes do not display sex-differential gene expression in CNS tissues (see above) and that *fru P1* (transcripts from *P1* promoter produce FRU^M^) acts predominately in the nervous system [4,10,63,64], we propose that *fru P1* functions to specify male-specific behaviors by modulating expression of genes that are expressed in both male and female head tissue. Given the modulatory nature of *cis*-regulatory promoter regions, these genes are likely to be regulated in males and females by different transcription factor sets, in different cells, and thus may have the same overall expression levels in males and females, but have different spatial patterns. Thus, we do not expect genes regulated by FRU^M^ activity to be male-specific or enriched, but we do expect them to show differential expression when comparing expression in *fru P1* mutant males to wild-type males.

Accordingly, we identified a set of genes regulated by FRU^M^, by comparing gene expression in adult heads, between males from two different wild-type strains (CS and Berlin) versus males from two different *fru P1* allele combinations (*fru^440/p14^* and *fru^w12/cham5^*; see Figure 1). The *fru P1* allele combinations are null/strong loss-of-function allele combinations for *fru P1* transcript classes [6,65,9]. We note that one of these *fru* allele combinations (*fru^440/p14^*) also removes *fru P2* transcripts and some expression differences we report may be due to the loss of transcripts produced from the *P2* promoter. Additionally, the *fru P1* mutant allele combinations are transheterozygotes for deficiency chromosomes and so may be hemizygous for loci adjacent to *fru*. By requiring that the genes show expression differences between two wild-type and two *fru P1* allele combinations, we can reduce the likelihood of identifying genes that are differentially expressed because of differences in strain background that affect transcription, which can be substantial [66,67], or because of the other genes located near the *fru* locus that are removed by each *fru* deficiency combination used here [6,65].

We combined the data from the two wild-type male versus *fru P1* male comparisons for statistical analyses, with the rationale that differentially expressed genes should show the same direction of change in the two mutant combinations as compared to wild type. In our identification of the DSX set, we used three genotype comparisons and thus used stringent statistical criteria, as a gene had to pass three different FDR tests. For the *fru P1* comparisons, we only used one comparison to define genes regulated by FRU^M^ (wild type versus *fru P1* males). Thus, we used a more stringent FDR cutoff (*q <* 0.05), as well as FC > 2, as criteria. This resulted in a list of 90 genes, of which 54 and 36 showed higher expression in *fru P1* males and wild-type males, respectively (Table S7). This set of *fru P1*-regulated genes will hereafter be referred to as the “FRU^M^ head set”. As confirmation of the microarray expression data, *fruitless* was identified in the FRU^M^ head set, showing higher expression in wild-type males than in *fru P1* males.

### Functional Categories That Are Enriched in the FRU^M^ Head Set

Using DAVID, we identified functional categories that are enriched in the FRU^M^ set; 88/90 genes were used in this analysis, as two of the 90 FRU^M^ head set genes did not have GenBank accession numbers (Table S7; *p* < 0.05). The FRU^M^ head set was significantly enriched for functional categories involved in sensory perception, including the categories “response to light” (*p* < 0.019) and “response to both physical and chemical stimuli” (*p* < 3.9E-3). These over-represented functional categories are consistent with recent reports that *fru P1* is localized not only to the CNS [64], but also the peripheral nervous system (PNS) [10,63]. We also report significant enrichment (*p* < 2.9E-3) for cytochrome p450 genes, which are involved in steroid metabolism and xenobiotic detoxification. Cytochrome p450 genes are known to have sex-differential expression in the fat body [23,31,36]. Male courtship behavior has been shown to be disrupted via feminization of the fat body [38]. Perhaps one way that FRU^M^ regulates behavior is by modulating steroid metabolism and affecting circulation levels of steroid hormones [38]. This may occur by influencing gene expression in non-neuronal tissues indirectly, as *fru P1* has not been detected in fat body tissues using various techniques [64,65,68]. Additional possibilities are that subsets of the cytochrome p450 genes are expressed in the nervous system, or alternatively, *fru P1* may be expressed at low levels in fat body tissues.

Other genes that that are enriched in our FRU^M^ head set are those involved in circadian rhythm processes (*p* < 2.4E-5). Other studies have shown that the establishment and maintenance of neurons involved in circadian processing impacts the number of *fru P1*-expressing cells. For example, the number of *fru*-expressing neurons decreases when toxic proteins are expressed in *timeless* (*tim*) cells, suggesting the possibility that *tim*-expressing cells might synapse on *fru*-expressing cells and that this is required for maintenance and modulation of a subset of *fru*-expressing cells [36]. Additionally, *neuropeptide F* (*npf*)-expressing cells are a subset of *tim*-expressing neurons, and *fru P1* brains show reduced *npf* expression [24]. *CG9377* is among several genes encoding serine-type peptidases identified as having differential expression as a consequence of *fru P1* expression, and shows circadian-dependent expression levels [57,58]. The enrichment of genes that are under circadian regulation that are also regulated by *fru P1*, supports the idea that there is a direct molecular tie between regulating circadian rhythms and courtship behaviors, as would be expected for a behavior that displays periodicity based on the circadian clock.

### Genome-Wide Identification of Genes Regulated by FRU^M^ in Adult CNS Tissue

To identify genes that underlie behavior, and to distinguish between genes that show differential expression as a consequence of *fru P1* expression in the CNS from those expressed in other tissues of the adult head, we performed microarray experiments using RNA extracted from dissected CNS tissues. Microarray experiments were performed using RNA extracted from both dissected brains and ventral nerve cords from wild-type males and *fru P1* males (see Materials and Methods). We identified 26 genes (hereafter called the “FRU^M^ CNS set”) with significant differential expression, of which 17 and 9 showed higher expression in *fru P1* male and wild-type male dissected CNS tissue, respectively (Table S8). While the feature for the *fru* transcript showed borderline FDR significance, a modified Student's *t*-test showed a significant difference in expression (*p* < 0.004), and in all six experiments, the data from this *fru* array element showed higher expression in males with an average FC > 1.5.

We identified *capability* (*capa*) in our FRU^M^ CNS set with higher expression in dissected CNS tissues of wild-type males than *fru P1* males, thus *capa* may be induced by FRU^M^. *capa* is a gene predicted to be involved in neuropeptide hormone signaling and ion transport [69–71]. We validated the microarray expression results using RT-PCR and report significantly higher expression in the dissected CNS tissue of males than *fru P1* males (∼1.4 fold, *p* < 9.2E-4; Figure 4C).

Using DAVID, we identified “ion transport” (*p* < 0.02) and “establishment of localization” (*p* < 0.03) as functional categories over-represented in the FRU^M^ CNS set. The FRU^M^ CNS set includes *CG8713* and *CG11710*, genes whose products are inferred to play a role in potassium ion transport and transmission of nerve impulses, and to have transcription cofactor activity, respectively [72,73]. Additionally, the FRU^M^ CNS set includes *capa* (see above), and *resistant to dieldrin*, a gene shown to have gamma-aminobutyric acid (GABA-A) receptor and neurotransmitter activity [74]. In light of recent studies that showed that the *fru P1*-expressing circuit is present at a morphologically indistinct level in both males and females, [10,63,75] with the exception of small differences in cell number [75], the functional categories that we identified are consistent with FRU^M^ playing a role in neurophysiology or fine-scale connectivity, as has been suggested [10,63].

### FRU^M^ Regulates Different Sets of Genes in Head Tissues as Compared to CNS Tissues

Our data showed substantially more genes with significant differential expression between wild-type males and *fru P1* males when we assayed RNA extracted from adult heads, versus RNA extracted from dissected brains and ventral nerve cords. This was also the case when we analyzed sex-differential expression between RNA from males and females from head tissues, compared to RNA derived from CNS tissues. We have the power to detect 99.5% of true positives and report a high Pearson correlation (*r* ^2^ = 0.96) among all FRU^M^ CNS set experiments (see Materials and Methods), demonstrating that the reason we identified fewer genes in the FRU^M^ CNS set is not because of a technical problem. This suggests that the majority of genes that were identified as regulated by FRU^M^ in the adult head are expressed outside of the brain and ventral nerve cord. Given this gene list, these genes are most likely expressed in the fat body and other PNS tissues or perhaps glial cells.

There have been several studies that implicate the fat body as playing an important role in behaviors, by potentially producing secreted circulating proteins. One such gene, *takeout* (*to*), was shown to encode a secreted signaling molecule that can be found in the hemolymph and is regulated by the sex hierarchy [23]. TO has much higher levels in males as compared to females, and *to* mutants show a reduced courtship index [23,38]. On the basis of these studies it was proposed that TO may be a fat body–diffusible factor that serves hemolymph-brain communication to help regulate courtship activity [23,38].

As noted above, other studies have identified many sex-differentially expressed genes in adult head tissue [23,31,36], however previous studies did not distinguish between the numbers of genes that were differentially expressed in the CNS versus other tissues of the head. Furthermore, those studies did not determine the role of *fru P1* in establishing gene expression levels in these other head tissues, as it is thought that the primary role of FRU^M^ is in the nervous system. Our expression results identified hundreds of genes (754) with significant sex-biased expression in the adult head that are not significantly sex differentially expressed in wild-type CNS tissues. Although our stringently defined FRU^M^ head set contains 90 genes (*q* < 0.05, FC > 2), by relaxing the statistical stringency, we have identified over 1,000 additional genes with significant differential expression in *fru P1* versus wild-type adult male heads that are not significantly differentially expressed in *fru P1* versus wild-type male CNS tissue comparisons (*q <* 0.15, 1,554 genes, unpublished data). Furthermore, none of the six genes identified as having significant differential expression in both the FRU^M^ adult head and FRU^M^ CNS datasets were identified as having significant sex-biased expression in either the wild-type adult head, or the wild-type dissected CNS datasets. Taken together, our results suggest the possibility of independent sex-specific and *fru P1*-specific regulation of fat body genes. Furthermore, these studies bolster the previously suggested idea that *fru P1* likely influences adult-male fat body–gene expression [23,76].

Given the complexity of male courtship behavior and the essential role of FRU^M^ in specifying this behavior, it is surprising that a greater number of genes that are regulated downstream of FRU^M^ activity were not identified in the CNS. FRU^M^ is expressed in ∼1,200–1,300 adult CNS cells [10,64]. Although we detect differences in *fru P1* levels in our experiments, perhaps FRU^M^ targets are expressed at lower levels. It is also possible that FRU^M^ targets are not regulated by *fru P1* throughout the circuit, or are under the regulation of other transcription factors in other cells of the CNS, thus making it difficult to detect expression level differences in the comparisons we have made. Future studies analyzing gene expression in subsets of FRU^M^-expressing cells will address this concern.

### *dpr* and *dpr*-Family Members Are Transcriptionally Regulated by FRU^M^ in the Adult Head

Given the observation that both males and females have nearly the same number of homologously positioned *fru P1* neurons and these neurons have similar axonal projection patterns [10,63,75], it has been proposed that FRU^M^ in males may either modulate neuronal activity or fine-scale connectivity to bring about the potential for male courtship behaviors [10,63]. Thus, we searched our dataset to find genes whose functions are consistent with these roles to further analyze and chose *defective proboscis extension response* (*dpr*). *dpr* was identified in a genetic screen for genes that underlie the behavioral response of proboscis removal from a high salt solution [77]. *dpr* is the founding member of a family of genes encoding predicted cell adhesion molecules that contain two Ig domains. *Drosophila* Ig-containing proteins have been classified as either secreted or membrane-bound ligands, cell adhesion molecules, or transmembrane receptors. Since the cytoplasmic domain of DPR is only 75 amino acids, DPR is inferred to be a cell adhesion molecule, or a membrane-bound ligand. Previous studies identified a family of 20 *dpr*-related genes (*dpr1*–*dpr20*) that encode predicted proteins with 30%–52% amino acid similarity within both Ig domains of DPR [77]. Nineteen of the 20 *dpr*-family genes were represented as features on our microarrays.

Several members of the *dpr* family appear to be regulated by FRU^M^ (Figure 4B). Statistical analyses of our array data using FDR identified five *dpr*-family genes with significantly higher expression in wild-type males than *fru P1* males (*q* < 0.1). The observation that five out of 19 *dpr*-family members are differentially expressed is significantly greater than what is expected, on the basis of the number of *dpr* genes in the genome (*p* < 0.01, hypergeometric test). We furthered these analyses by determining if there is an enrichment of *dpr*-family members in genes that are regulated by FRU^M^, by examining the *fru^440/p14^* and *fru^w12/ cham5^* versus wild-type male comparisons independently. Here we reasoned that differences in the *fru* allele combinations might affect *dpr-*family gene expression differently. We found eight and two additional *dpr*-family genes with significantly higher expression in wild-type males than *fru^4–40/p14^* males and *fru^w12/cham5^* males, respectively. Overall, 15 of the 19 *dpr*-family genes showed significantly higher expression in males than either *fru^440/p14^* or *fru^w12/cham5^*, or both. This is significantly greater than what is expected, on the basis of the number of *dpr-*family genes in the genome (*p* < 1.0E-4, hypergeometric test), suggesting this family of genes may be regulated in a similar manner by FRU^M^.

Here we focus on *dpr*, which showed higher expression in wild type than *fru P1* head tissues (∼1.3 fold, *q* < 0.036). We confirmed our microarray results (Figure 4B) by RT-PCR and found that *dpr* is significantly higher (∼4.4 fold, *p* < 0.05) in wild-type males as compared to *fru P1* mutants.

### *dpr* and FRU^M^ Overlap in Expression in the Adult Brain and Ventral Nerve Cord

We next determined if FRU^M^ is expressed in subsets of *dpr*-expressing cells, to ascertain if it is possible that direct regulation of *dpr* by FRU^M^ could account for reduced *dpr* expression levels in the *fru P1* mutant. We visualized FRU^M^ and *dpr*-expressing cells using immunohistochemistry. *dpr*-expressing cells were detected using an upstream activating sequence (UAS) directing expression of nuclear green fluorescent protein (GFP) reporter, driven by GAL4 that is inserted in the *dpr* locus (hereafter called *dpr-GAL4*). The *dpr-GAL4* expression pattern has been previously characterized and shown to recapitulate most endogenous *dpr* expression [77].

**Figure 6:**
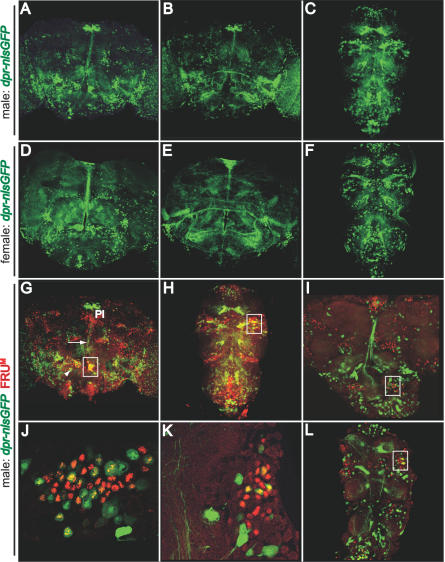
*dpr* Expression in the CNS Colocalizes with FRU^M^ in Adults and 48-h Pupae and Is Not Sexually Dimorphic In all images, *dpr-GAL4; UAS-nlsGFP* (hereafter called *dpr* expression) is shown in green. (A–C) *dpr* expression in the 0–24-h adult male CNS. (A) Anterior brain z-section. (B) Posterior brain z-section. (C) Anterior ventral nerve cord z-section. (D–F) *dpr* expression in the 0–24 h adult female CNS. (D) Anterior brain z-section. (E) Posterior brain z-section. (F) Anterior ventral nerve cord z-section. (G–H) *dpr* and FRU^M^ (red, immunohistochemistry) expression in the 0–24-h adult male CNS. (G) *dpr* and FRU^M^ expression in the anterior male brain. Colocalized expression of *dpr* (green) and FRU^M^ (red) is observed in the median bundle neurons (white box). *dpr* is expressed in cells in the axonal tract extending from the median bundle (arrow), and in the pars intercerebralis (PI). (H) *dpr* and FRU^M^ expression in the 0–24-h adult male ventral nerve cord. Colocalized expression of *dpr* (green) and FRU^M^ (red) is observed in the first thoracic ganglion (white box). (J) *dpr* (green) and FRU^M^ (red) expression in 40× magnification of median bundle neuron region (white box in [G], independent brain). (K) *dpr* (green) and FRU^M^ (red) expression in 40× magnification of first thoracic ganglion region (white box in [H], independent VNC). *dpr* and FRU^M^ expression in the 48-h pupal male brain (I) and ventral nerve cord (L). Z-section thickness was ∼1 μm for (A–I), (L), and ∼0.5 μm for 40× images (J–K). doi:0.1371/journal.pgen.0030216.g006

We observed colocalization of FRU^M^ and *dpr-*expression in the nuclei of ten to 15 neurons that are below the median bundle axon tracts (Figure 6J). When we examined the *dpr-*expressing cells projection patterns (Figure 6G, arrow), it appears that they are part of the ascending median bundle neurons. Subsets of neurons in the ascending median bundle were previously described as playing a role in courtship gating, the process that controls the timing and the sequence of progression through the courtship ritual [78]. The nuclei of these ascending median bundle neurons are in the subesophegial ganglion (SOG) region; the SOG contains neurons that are innervated by primary gustatory sensory neurons that extend from the proboscis to the brain [79]. The SOG is also the termination site of labellar and tarsal gustatory neurons [80–83]. We also observed colocalization of FRU^M^ and *dpr*-expressing cells in three to six cells of the first thoracic segment in the ventral nerve cord (Figure 6H and 6K). This region of the CNS has been implicated in wing song formation during courtship performance [84,85], though further experimentation is required to ascertain if altered *dpr* expression in *fru P1* mutants affects courtship song.

We observed close positioning of many of the *dpr*-expressing cells with FRU^M^-expressing cells in the SOG and the pars intercerebralis (PI) region (Figure 6G, labeled “PI”), raising the possibility that *fru P1* influences *dpr* expression via synaptic connections and neuronal activity of FRU^M^-expressing cells, rather than directly regulating transcription. The PI region is known to contain large neurosecretory cells. It has been shown that ablation or perturbation of four neurons in the pars intercerebralis region, leads to increased courtship activities [86]. Perhaps, *dpr*-expressing cells in the PI are involved in modulating courtship activity rate and latency, which is consistent with the behavioral phenotypes we observed for *dpr* (see below).

Overall, given that some *dpr*-expressing cells overlap with *fru P1*-expressing cells, it is possible that FRU^M^ directly regulates levels of *dpr* expression in subsets of *dpr*-expressing cells. Alternatively, wild-type activity of *fru P1*-expressing neurons may be important for the maintenance of *dpr* expression levels, especially in *dpr*-expressing cells that are in close proximity to *fru P1*-expressing cells, like those in the SOG and PI regions of the brain.

### *dpr* Is Expressed in the PNS in Regions Known to Be Important for Courtship Performance

Given the observation that *fru P1* is expressed in the PNS [10,63], we wanted to determine if FRU^M^ plays a role in regulating *dpr* expression in the PNS. However, because FRU^M^ and *dpr*-expressing cells in the PNS are both only detectable using the GAL4 system, we were limited in our ability to determine colocalization [10,63,75] and so here describe our observation of *dpr* expression in the PNS. We observed *dpr*-expressing cells in the forelegs and proboscis (Figure S1), consistent with previous descriptions [77], and near previously reported *fru P1*-expressing cells [10,63]. Thus, an intriguing possibility is that *dpr* may play a role in both primary sensory cells, as well as in cells involved in higher order processing of gustatory information, such as those we detect in the SOG region.

### *dpr*-Expressing Cells Are Not Sexually Dimorphic in the CNS of Adult or 48-h Pupae

Given that *dpr* was identified as being regulated by FRU^M^, we sought to determine if the number and pattern of *dpr*-expressing cells is sexually dimorphic, in the adult or pupal CNS. In the adult and pupal male brain and ventral nerve cord (Figure 6A–6C) we observed a broad distribution of *dpr-*expressing cells throughout the midbrain, optic lobes, and ventral nerve cord, which is consistent with previous DPR expression studies in adult male head sections [77]. We did not observe any major differences in *dpr-*expression patterns at the gross morphological level between adult (Figure 6D–6F) or 48-h pupal male and female brains and ventral nerve cords (compare Figure 6A and 6C to Figure 6I and 6L), though quantitative differences are difficult to detect using the GFP reporter. Possibly, other non–sex-specific transcription factors establish the *dpr* spatial expression patterns, and in males FRU^M^ is required to maintain the levels of this expression. Alternatively, normal transcription levels of *dpr* in males may require *fru P1*-expressing cells to have wild-type neuronal activity, and the effects on *dpr* expression may be indirect. In females, other transcription programs/neuronal activity would be needed to maintain the *dpr* expression pattern

### *dpr* Mutations and Reduced *fru P1* in *dpr*-Expressing Cells Cause Courtship Defects

To determine if *dpr* and *fru P1* expression in *dpr*-expressing cells have a role in courtship behavior, we performed courtship analyses on *dpr* mutants and mutants in which the *fru P1* transcript is reduced in *dpr*-expressing cells. The *fru P1* transcript was reduced using a UAS-RNAi transgene called *fru P1-RNAi* [78], with expression driven by a *dpr-GAL4* transgene*.* To account for any possible background genetic effects, all transgenic strains described here were backcrossed to the same strain of *w*, CS flies (see Materials and Methods). The *dpr-GAL4* P-element insertion causes a loss-of-function mutation at the *dpr* locus [77].

### *dpr* Mutations and Reduced *fru P1* in *dpr*-Expressing Cells Lead to Reduced Courtship Latency as Assayed by First Wing Extension

**Figure 7:**
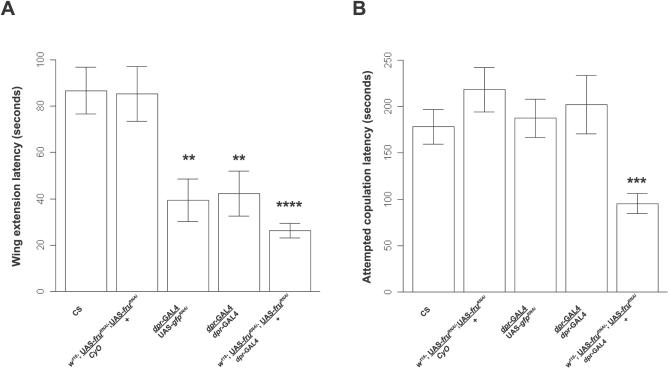
*dpr* Mutant Males Show Reduced Courtship Latency (A) Time (s) to first wing extension for CS male flies (*n* = 30, mean = 86.7, SE = 9.9) is significantly different (one-tailed *t*-test) than *dpr-GAL4* (*n* = 26, mean = 42.3, SE = 9.6) (*p* < 2.95E-5), *dpr-GAL4*;*UAS*-*gfp* ^RNAi^ (*n* = 50, mean = 39.4, SE = 9.3) (*p* < 1.73E-8), and *dpr-GAL4;UAS*-*fru* ^RNAi^ (*n* = 66, mean = 26.2, SE = 3.1) (*p* < 3.00E-10) flies. Also, when compared to *UAS*-*fru* ^RNAi^ (*n* = 38, mean = 85.3, SE = 11.7) male control flies, the time to first wing extension is significantly reduced in *dpr-GAL4* (*p* < 2.21E-4), *dpr-GAL4;UAS*-*gfp* ^RNAi^ (*p* < 2.28E-7), and *dpr-GAL4;UAS*-*fru* ^RNAi^ (*p* < 2.71E-9) flies. (B) The time to first attempted copulation (s) is significantly reduced in *dpr-GAL4*;*UAS-fru* ^RNAi^ (*n* = 63, mean = 95.3, SE = 10.8) flies compared to CS (*n* = 27, mean = 178.3, SE = 18.3) (*p <* 3.20E-5), UAS-*fru* ^RNAi^ (*n* = 33, mean = 218.5, SE=23.6) (*p <* 1.03E-6), *dpr-GAL4;UAS-gfp* ^RNAi^ (*n* = 45, mean = 187.5, SE = 20.9) (*p* < 3.41E-5), and *dpr-GAL4* (*n* = 24, mean = 202.2, SE = 31.1) (*p* < 1.45E-4) male flies. Error bars represent SE. Mann-Whitney rank-sum test: **, *p* < 1.0E-3; ***, *p* < 1.0E-4; ****, *p* < 1.0E-8. doi:0.1371/journal.pgen.0030216.g007

We quantified the amount of time it takes for a male to initiate wing extension, one of the steps in the male courtship ritual that can be reliably assayed. We observed a significantly reduced time to first wing extension in homozygous *dpr-GAL4* mutant males, as compared to wild-type males, and *UAS-fru P1-RNAi* transgene males (Figure 7A). Interestingly, we also saw a significant reduction in time to first wing extension in males that are transheterozygous for the *dpr*-*GAL4* allele and a *UAS-GFP RNAi* transgene, suggesting that the *dpr-GAL4* allele is a dominant allele with respect to initiation of wing extension or there is a nonspecific effect on wing extension due to the expression of the *GFP*-*RNAi* transgene. We observed an even greater reduction in time to first wing extension in *dpr-GAL4; fru P1*-*RNAi* males, compared to all the other male genotypes, suggesting that both *dpr* and *fru P1* expression in *dpr*-expressing cells are important for the wild-type timing of wing extension initiation.

### Reduced *fru P1* in *dpr*-Expressing Cells Lead to Reduced Time to Attempted Copulation

We next quantified the time to first attempted copulation and observed a significant reduction in the amount of time it takes *dpr-GAL4*; *fru P1-RNAi* transgene males (95.3 +/− 10.8 s, Figure 7B), as compared to all the other male genotypes we assayed. No significant differences in time to attempted first copulation were observed in males homozygous or heterozygous for *dpr-GAL4*, as was observed for wing extension. This suggests that the *dpr-GAL4* mutation had an effect on courtship timing up through wing song, but that abrogating *fru P1* function had an additional effect on the gating of courtship progression through to attempted copulation.

Furthermore, a higher percentage of *dpr-GAL4*; *fru P1-RNAi* male flies performed wing extension (WE) and attempted copulation (AC) (WE: *n* = 66, 100% and AC: *n* = 63, 95%, respectively) within the allotted time, as compared to wild-type males (WE: *n* = 30, 91% and AC: *n* = 27, 82%, respectively), suggesting that the reduction of *fru P1* expression in *dpr*-expressing cells manifests in quicker initiation of courtship activity throughout several stages of the courtship ritual. Given that *dpr-GAL4; fru P1-RNAi* males are heterozygous for the *dpr-GAL4* allele, it is possible that some of these phenotypes are an additive effect of removing *dpr* function and removing *fru P1* function.

### *dpr-GAL4*; *fru P1-RNAi* and *p52a-GAL4; fru P1*-RNAi Males Display Similar Courtship Phenotypes

The *dpr-GAL4*; *fru P1-RNAi* courtship phenotype is consistent with what was observed when *fru P1* transcript was reduced in the ascending median bundle neurons using a different GAL4 driver called *p52a-GAL4* [78]. *p52a-GAL4; fru P1-RNAi* mutant males courted females much more rapidly; <10 s for latency for courtship initiation, versus ∼70 s in wild-type CS flies, and ∼80 s for other control flies [78].

In addition, under wild-type conditions, initiation of courtship by one male towards a female will delay the initiation of courtship of a second male towards the same female [78]. In contrast, multiple *p52a-GAL4; fru P1-RNAi* mutant males were shown to immediately court and attempt copulation with a female, suggesting that inhibitory cues from other males or male–female pairs act through the ascending median bundle neurons [78]. We also observed this effect, as multiple *dpr-GAL4; fru P1-RNAi* males courted females without delay (unpublished data). We observe this phenotype only when the *dpr-GAL4; fru P1-RNAi* flies were raised at 29 °C, but not at 25 °C, suggesting the *fru P1-RNAi* transgene is more effective at 29 °C than at 25 °C. Taken together, the courtship phenotypes associated with reducing *fru P1* in the ascending median bundle suggest that the ascending median bundle neurons are important for timing of courtship progression. Besides reduced latency in courtship initiation and attempted copulation, *dpr-GAL4* and *dpr-GAL4; fru P1-RNAi* males appear normal morphologically and are not sterile. Additionally, these flies do not appear to have any mate-recognition problems, as they do not show any male–male chaining behavior.

### *dpr-GAL4; fru P1-RNAi,* and *dpr-GAL4* Males Do Not Show an Increase in General Locomotor Activity

To determine if the reduction in time to WE and AC in *dpr-GAL4; fru P1-RNAi,* and *dpr-GAL4* strains was due to a specific effect on courtship behaviors versus a nonspecific effect of increased locomotor activity, we assayed activity of these flies compared to controls (Figure S2). Here we observed a reduction in locomotor activity in the *dpr-GAL4; fru P1-RNAi,* and *dpr-GAL4* strains, compared to controls. During the time we performed our courtship assays the differences were not as robust as other times of the day (Zeitberger Time [ZT] 5–9). This suggests that the increased courtship activity we observed in *dpr* strains was not due to nonspecific increases in general locomotor activity. We cannot rule out that decreased locomotor activity results in increased courtship. However, it has been suggested that decreased locomotor activity is associated with increased latency to copulation [87], and that peak mating frequency occurs at times when flies are most active [62], suggesting that the courtship phenotypes we observed in the *dpr* mutants were not due to decreased locomotor activity.

### Conclusions

In this study we used microarray approaches to identify and describe genes that are sex differentially expressed and/or regulated by DSX (54 genes) and FRU^M^ in adult head (90 genes) and adult CNS tissues (26 genes). On the basis of these large datasets, we have described new models for DSX regulation of gene expression. One of the most striking observations from these studies is that the majority of genes that display sex-differential expression in the adult head are likely expressed in tissues outside of nervous tissue, such as those of the adult fat body. Furthermore, a large number of genes that are regulated by FRU^M^ are also expressed outside the CNS and appear to also be expressed in adult fat body. These results, together with recent work from other groups, suggest that modulation of adult behaviors including reproductive [38], feeding [88], aggressive [89], and circadian behaviors [36] is achieved through the activity of the fat body, perhaps through secreted molecules that affect neuronal function. In the future, functional analyses of genes expressed in the fat body will provide further insight into how this tissue influences sex-specific behavioral activities. We focused our functional analyses on male-biased genes that are expressed in the CNS, but many of the genes we identified may underlie the potential for other sexually dimorphic behavioral activities, including female receptivity, egg laying, feeding behaviors, circadian behavior, and aggression. Here we provide molecular inroads for the future study of these behaviors.

## Materials and Methods

### Drosophila stocks.

All stocks were grown using standard conditions at 25 °C unless otherwise noted. Wild-type stocks were CS, Berlin, and w; Berlin. Chromosomally XX, *tra* pseudomales are the genotype *y, w, P [w^+cM^, ubi-gfp]/w; tra^1^/ Df (3L) st-j7.* For the following strains, chromosomal sex appears in parentheses. Chromosomally XX, *dsx^D^* pseudomales are the genotype *y, w, P [w^+cM^, ubi-gfp]/w; dsx^D^, Sb/dsx^m+r15^* (XX). *dsx* intersexual animals are the genotypes *y, w, P [w^+cM^, ubi-gfp]; dsx^d+r3^/dsx^m+r15^* (XX) and *dsx^d+r3^/dsx^m+r15^* (XY). The genotypes of *fru P1* males are *fru^4–40^/fru^p14^* (XY) and *w; fru^w12^/fru^ cham5^* (XY). The *dpr-GAL4* [77] line was kindly provided by C. Montell (The Johns Hopkins University School of Medicine, Baltimore, MD, United States). The *UAS-fru^RNAi^* line *(w; P [w^+cM^,UAS-fru^RNAi^]/CyO; P[w^+cM^,UAS-fru^RNAi^]/ P[w^+cM^,UAS-fru^RNAi^])* was kindly provided by D. Manoli in the laboratory of B. Baker, (Stanford University, Stanford, CA, United States). All strains used in courtship assays were outcrossed for six generations to w, CS kindly provided by D. Guarnieri, in the laboratory of U. Heberlein (University of California at San Francisco, San Francisco, CA, United States).

### Microarrays.

We employed a two-color DNA microarray approach [90], using glass-slide arrays spotted with 15,158 oligonucleotide probes representing all known and predicted open-reading frames, based on release 4.1 of the D. melanogaster genome. The long oligonucleotide sequences were designed by the International Drosophila Array Consortium (INDAC) using a custom implementation of OligoArray2 [91]. The oligonucleotides were designed with size ranges between 65–69 nucleotides, a minimal Tm window, bias towards the 3′-ends of transcripts, and minimal sequence similarity to other genes [92]. The oligonucleotides were synthesized by Illumina. The oligonucleotide sequences can be downloaded from Flymine: http://www.flymine.org/release-5.0/aspect.do?name=INDAC. Microarrays were printed in the laboratory of Eric Johnson (University of Oregon, Eugene, OR, United States) using slides coated with aldehyde chemistry and were postprocessed using the Nunc SuperChip Aldehyde protocol (Thermo Fisher Scientific).

### RNA extraction and target preparation.

For experiments in which RNA was extracted from adult head tissues, flies were collected under CO_2_ anesthesia (ZT 2) and allowed 8 h (ZT 10) to recover to minimize identifying genes induced by exposure to CO_2_, before being snap frozen in liquid nitrogen. All collections were timed such that all adult flies were between 8–24 h posteclosion when snap frozen. For experiments performed using RNA from dissected CNS tissues, animals were lightly anesthetized at the time of dissection and dissections were performed rapidly. Total RNA was extracted from fly heads or dissected CNS tissues by homogenization and extraction using TRIzol reagent (Invitrogen). All experiments included dye-flip hybridizations, to minimize identifying genes because of differences in dye incorporation, with one half of the comparisons labeled in one dye orientation and the other half the other dye orientation.

For experiments using RNA derived from head tissues, total RNA (20 μg) was used as template to make cDNA in the presence of Cy-labeled nucleotides (direct labeling). Direct labeling was achieved through a 2-h reverse transcription reaction at 42 °C. Final concentrations are indicated in parentheses: oligo dT primer (Operon, 3.75 μM), dithiothreitol (Invitrogen, 10 mM), First Strand Buffer (Invitrogen, 1×), dNTPs minus dTTP (Invitrogen, 0.5 μM), dTTP (Invitrogen, 50 nM), Cy-labeled dUTP (Perkin-Elmer, 0.625 nM), and Superscript II reverse transcriptase (Invitrogen, 10 U/μL). The reaction was quenched using NaOH (167 mM) and EDTA (83 mM) and purified using the Qiaquick PCR purification kit (Qiagen). Targets were dried and resuspended in formamide (25 μM), 3 M NaCl, and 0.3 M sodium citrate buffer (SSC, 3.3×), SDS (1.1%), Denhardts (5.56×), and polyA solution (8.88 μM), and boiled for 2 min. The resuspended labeled cDNA was applied to the microarrays and allowed to hybridize 16–18 h at 42 °C. Slides were washed in 300 ml of a 1.5% SDS, 1× SSC solution for 5 min, followed by a 5-min wash in 0.2 × SSC, and twice for 10 min in dH_2_O, spun dry, and scanned with 635-nm and 532-nm lasers using a Genepix 4100A scanner (Axon Instruments).

cRNA probes were made from RNA derived from CNS tissue using the Amino Allyl MessageAmp II aRNA Amplification Kit (Ambion). All experiments were performed using at least four independent biological samples of ∼100 fly heads each for head experiments and ∼20 dissected brains and VNCs for CNS experiments; for the experiments comparing wild-type male versus female and fru P1 male versus wild-type male, eight and six independent biological samples were used, respectively.

### Microarray data analyses.

Poor quality features, identified by visualization of the scanned microarray, were removed from the raw data before analysis using Genepix software. Resulting Genepix data were analyzed using the R Bioconductor software package [93]. Filtering identified and kept features containing >75% of total pixels above 1 standard deviation above background for further study. Data were normalized using the Bioconductor lowess method [94]. All analyses were performed on log-transformed ratio values. To adjust for multiple testing, the positive FDR [26] was calculated for each gene using the Bioconductor FDR package to determine the significance of differences. All significance tests used a *q* < 0.15 for significance cutoff unless otherwise noted. Antilogarithm (base 2) was applied to the data to obtain FC values.

Post hoc power calculations were based on a *t*-test using <0.05 to measure the difference between the means of two independent groups and were computed using the GPower3.0.3 program [95]. The effective size for power was calculated using the mean intensity and standard deviation of all the genes within the 50th percentile of genes with the least variance. Pearson correlation (*r* ^2^) was determined across all experiments using dye-flip intensity values for all genes with a calculated variance. The expression data are publicly available at http://www.ncbi.nlm.nih.gov/geo/.

### Wild-type male and female comparison.

An analysis of the combined data for CS and Berlin demonstrates that we have the power to detect 87.1% of gene expression differences (see “Microarray data analyses” in Materials and Methods). We calculated the Pearson correlation (*r* ^2^) between all hybridizations and report *r* ^2^ = 0.88 for these experiments, suggesting that we do not have a high amount of experimental variation between our replicates.

### *tra* pseudomale and female comparison.

Sex-biased, *tra-*independent genes were identified by considering only significant sex-biased genes that did not pass a *q* < 0.25 statistical cutoff and had data in all four *tra* versus wild-type female array replicates. Similarly, *tra*-regulated, but DSX- and FRU^M^- independent genes, were identified by considering only genes that were significant in the *tra* comparison; did not pass *q* < 0.25 for the *dsx^D^* comparison; had data for all four *dsx^D^* versus wild-type female array comparisons; did not pass *q* < 0.25 for the *fru P1* comparisons; and had data in at least six of the eight *fru P1* versus wild-type male array comparisons.

### Real-time PCR.

Total RNA was extracted using standard TRIzol protocols and treated with DNaseI (Ambion) and MgCl_2_. cDNA was synthesized following the SuperScript II Reverse Transcriptase protocol using Random Primers (Invitrogen). Real-time PCR was performed using a DNA Engine Opticon 2 detection system (BioRad), utilizing AmpliTaq Gold PCR Master Mix (Applied Biosystems) and SYBR Green (Applied Biosystems) fluorescence. The following primer pairs were used: cpn (5′-TGGAGCGACAGCCACTTCTG and 5′-GCAGACGTTGCTCCACCTGA), dpr (5′-CGCCAATTGGACACTGCAAA and 5′-GCTCGTGGGGTCCTTGCATA), capa (5′- CCACTGGCTTTCTTTTGGAA and 5′-AGTCTGCGCGACGGATTAG), and rp49 (5′- GCCAAACTGATGCTAGGC and 5′-CCACCTCCACTTCAGGATAC). Cycling was for 10 min at 94 °C, followed by 45 cycles of 94 °C for 20 s, 60 °C for 30 s, and 72 °C for 30 s. Each of four independent samples for each genotype, consisting of ∼1.2 μg RNA, was assayed in three technical replicates. Expression for each gene relative to *rp49* expression was calculated using Data Analysis for Real Time (DART) PCR [96]. *t*-Tests were used to determine the significance of differences between the genotypes assayed.

### Frozen section in situ hybridization.

Detection of cpn mRNA in frozen sections prepared from adults heads was performed as described by Goodwin et al. [65], using digoxigenin-labeled anti-sense and sense cpn cRNA probes, made from cDNA clone GH08002, available from the Berkeley Drosophila Genome Project (BDGP).

### Functional GO analyses.

The bioinformatics tool DAVID [44] was used to determine significant enrichment of known functional annotations within our gene lists. DAVID calculates the statistical probability for representation of genes within a given functional category for an input list relative to the total number of genes, within the same category for a background list. The background list consisted of 13,614 GenBank accession numbers representing each unique transcript on our arrays. Search parameters included all available categories from two GO ontologies, biological process (BP), and molecular function (MF). All significantly (*p* < 0.05) enriched functional groups within all levels of BP and MF were reported in the Supporting Information tables. We reported level four BP and MF for all genes without an enriched functional category. If level four BP and MF were not available, we reported level three. An additional literature search parameter was added to the DAVID analysis to achieve significant enrichment for the circadian and oxidative-stress functional categories.

### Immunohistochemistry.

*dpr-GAL4;UAS-nlsGFP* 0–24-h adult flies were dissected in 1× phosphate buffered saline (PBS), fixed in 4% paraformaldehyde, 1× PBS for 20 min and stained as described in Lee et al [64]. Here, brains and ventral nerve cords were stained using primary rat polyclonal FRU^M^ antisera (1:100) and secondary Cy5-conjugated goat anti-rat IgG and Cy3-conjugated rabbit anti-GFP IgG (1:500; Molecular Probes). We generated the FRU^M^ antisera (Josman Laboratories) against the 101 amino acid male-specific region, as described previously [64]. Adult peripheral tissues (forelegs, proboscis, antennae, and external genitalia) in 1× PBS were fixed, washed twice in 10 mM Tris-Cl, 150 mM NaCl, 0.05% Tween-20 buffer (TNT), and mounted in Vectashield mounting media (Vector Labs). Optical section stacks were obtained using a Zeiss LSM 5 Pascal confocal microscope and processed using Adobe Photoshop.

### Courtship assays.

Adult male flies were reared individually in vials and aged 4–6 d in a 12-h light: 12-h dark cycle. For courtship assays, a single male fly of each genotype was paired with a 4–6-d-old CS virgin female in a 10-mm diameter chamber and video recorded for 10 min. To account for any temperature or circadian effects, all courtship assays were performed at 22 °C, ∼60% humidity, and at ZT 5–9. Assays for control and experimental flies were performed on the same day. Courtship latency was considered the time until first wing extension. All courtship recordings were analyzed using Noldus software (Wageningen, Netherlands). Data were averaged, and standard error (SE) was determined for each genotype. Whitney-Mann (nonparametric) rank-sum tests were calculated to determine the significance of differences.

### Locomotor activity assays.

Three individual vials with food and containing 20 male flies of each genotype were assayed. Flies were entrained in a 12-h light: 12-h dark cycle for at least 4 d. Line crossings were counted in 30-min bins over a 48-h period using the *Drosophila* Activity Monitoring System (Trikinetics) in the same light:dark cycle as entrainment. Data were averaged, and SE was determined for each genotype.

## Supporting Information

Figure S1 *dpr-GAL4* Expression in the Forelegs and Proboscis Dissected *dpr-GAL4; UAS-nlsGFP* (A) forelegs and (B) proboscis (see Materials and Methods).
(1.6 MB AI).

Figure S2 *dpr* Mutant Males Show Reduced Locomotor Activity The mean number of line crossings for three independent groups of male flies (*n* = 20 per group, total of 60) of each genotype was calculated every 30 min over a 48-h light:dark cycle (see Materials and Methods). Tick marks along the *x*-axis represent 30-min increments. Boxes represent time flies spent in light (white) and dark (black). Blue diamonds, CS; black squares, *dpr-GAL4*; red triangles, *dpr-GAL4; UAS*-*fru* ^RNAi^.
(1.2 MB AI).

Table S1 Sex-Differential Genes List of 46 genes with significant differential expression (*q <* 0.15, FC > 2) between wild-type male and female adult heads. The white region contains genes with higher expression in females, and the gray region contains genes with higher expression in males. FC and *q*-values are reported. F/M indicates female over male microarray ratio values.
(17 KB XLS)

Table S2 Genes Downstream of *tra*
List of 117 genes that displayed significant differential expression between wild-type females and males and between wild-type females and *tra* pseudomales (one-tailed *t*-test). FCs are reported. The white region contains genes with higher expression in females, and the gray region contains genes with higher expression in males. The 54 asterisked genes were also identified in the DSX set. F/M and F/*tra* indicate female over male and female over *tra* microarray ratio values, respectively.
(24 KB XLS)

Table S3 Sex-Biased Genes Regulated in Opposite Manner by *tra*
List of 24 genes that displayed significant (*q* < 0.15) sex-biased and *tra*-dependent differential expression, but in opposite directions as observed in wild-type animals. FCs are reported. The white region contains genes with higher expression in females, and the gray region contains genes with higher expression in males. F/M and F/*tra* indicate female over male and female over *tra* microarray ratio values, respectively.
(15 KB XLS)

Table S4 DSX Set List of 54 genes that displayed significant differential expression between wild-type females and males, as well as between females compared to both *tra* pseudomales and *dsx^D^* pseudomales (one-tailed *t*-tests). FCs and functional annotations are reported (see Materials and Methods). The white region contains genes with higher expression in females, and the gray region contains genes with higher expression in males. F/M, F/*tra,* and F/*dsx^D^* indicates, female over male, female over *tra,* and female over *dsx^D^*, microarray ratio values, respectively.
(37 KB XLS)

Table S5 Genes Regulated by TRA, but Not Downstream of DSX or FRU^M^
List of 24 genes that showed significant differential expression between wild-type females and males, wild-type females and *tra* pseudomales (*q <* 0.15, one-tailed *t*-test), but are not regulated by DSX or FRU^M^ (see Materials and Methods). F/M, F/*tra,* F/*dsx^D^*, and *fru P1*/M indicates, female over male, female over *tra,* female over *dsx^D^*, and *fru P1* mutant male over male, microarray ratio values, respectively.
(22 KB XLS)

Table S6 Genes Regulated by Only One DSX Isoform List of 32 genes with significant differential expression (*q* < 0.25) in wild-type female versus male, female versus *tra* pseudomale, female versus *dsx^D^* pseudomale in 0–24-h adult heads. Top: 12 genes regulated by DSX^F^. The white region contains genes activated by DSX^F^, and the gray region contains genes repressed by DSX^F^. Bottom: 20 genes regulated by DSX^M^. The white region contains genes repressed by DSX^M^, and the gray region contains genes activated by DSX^M^. Columns contain FC ratio values from the microarray data: F/M, female over male; F/*tra*, female over *tra*; F/*dsx^D^*, female over *dsx^D^*; F/*dsx*, female over *dsx* null XX; M/*dsx*, male over *dsx* null XY. The symbols (+/−) in the first column are qualitative representations of activation (+) or repression (−), based on the microarray data.
(23 KB XLS)

Table S7 FRU Head Set List of 90 genes with significant differential expression (*q <* 0.05, FC > 2) between wild-type male and *fru P1* male adult heads. FCs and functional annotations are reported (see Materials and Methods). The white region contains genes with higher expression in *fru P1* males, and the gray region contains genes with higher expression in wild-type males. The six asterisked genes were also identified in the FRU CNS set. *fru P1*/M indicates *fru P1* mutant male over male microarray ratio values.
(45 KB XLS)

Table S8 FRU CNS Set List of 26 genes with significant differential expression between wild-type male and *fru P1* male adult dissected CNS tissue. FCs and functional annotations are reported (see Materials and Methods). The white region contains genes with higher expression in *fru P1* males, and the gray region contains genes with higher expression in wild-type males. The six asterisked genes were also identified in the FRU head set. *fru P1*/M indicates *fru P1* mutant male over male microarray ratio values.
(29 KB XLS)

## Abbreviations

AC: attempted copulation
BP: biological process
*capa*: *capability*
CNS: central nervous system
*cpn*: *calphotin*
CS: Canton Special
DAVID: Database for Annotation, Visualization, and Integrated Discovery
*dpr*: *defective proboscis extension response*
*dsx*: *doublesex*
DSX^F^: female-specific DSX isoform
DSX^M^: male-specific DSX isoform
FDR: False Discovery Rate
*fit*: *female-specific independent of transformer*
*fru*: *fruitless*
FRU^M^: males-pecific FRU isoform
*fru P1*: *fru* transcript class that is the product of the *P1* promoter
GFP: green fluorescent protein
MF: molecular function
PI: pars intercerebralis
PNS: peripheral nervous system
RT-PCR: real-time PCR
SE: standard error
SOG: subesophegial ganglion
*to*: *takeout*
*tra*: *transformer*
UAS: upstream activating sequence
WE: wing extension
*Yp*: *Yolk protein*
ZT: Zeitberger Time
