# Supplementary material for: Genomic and Functional Studies of Drosophila Sex Hierarchy Regulated Gene Expression in Adult Head and Nervous System Tissues
Source: PLoS Genet. 2007 Nov 23;3(11):e216. doi: 10.1371/journal.pgen.0030216 (PMC2082469; doi:10.1371/journal.pgen.0030216)

Supplemental Figure 2.

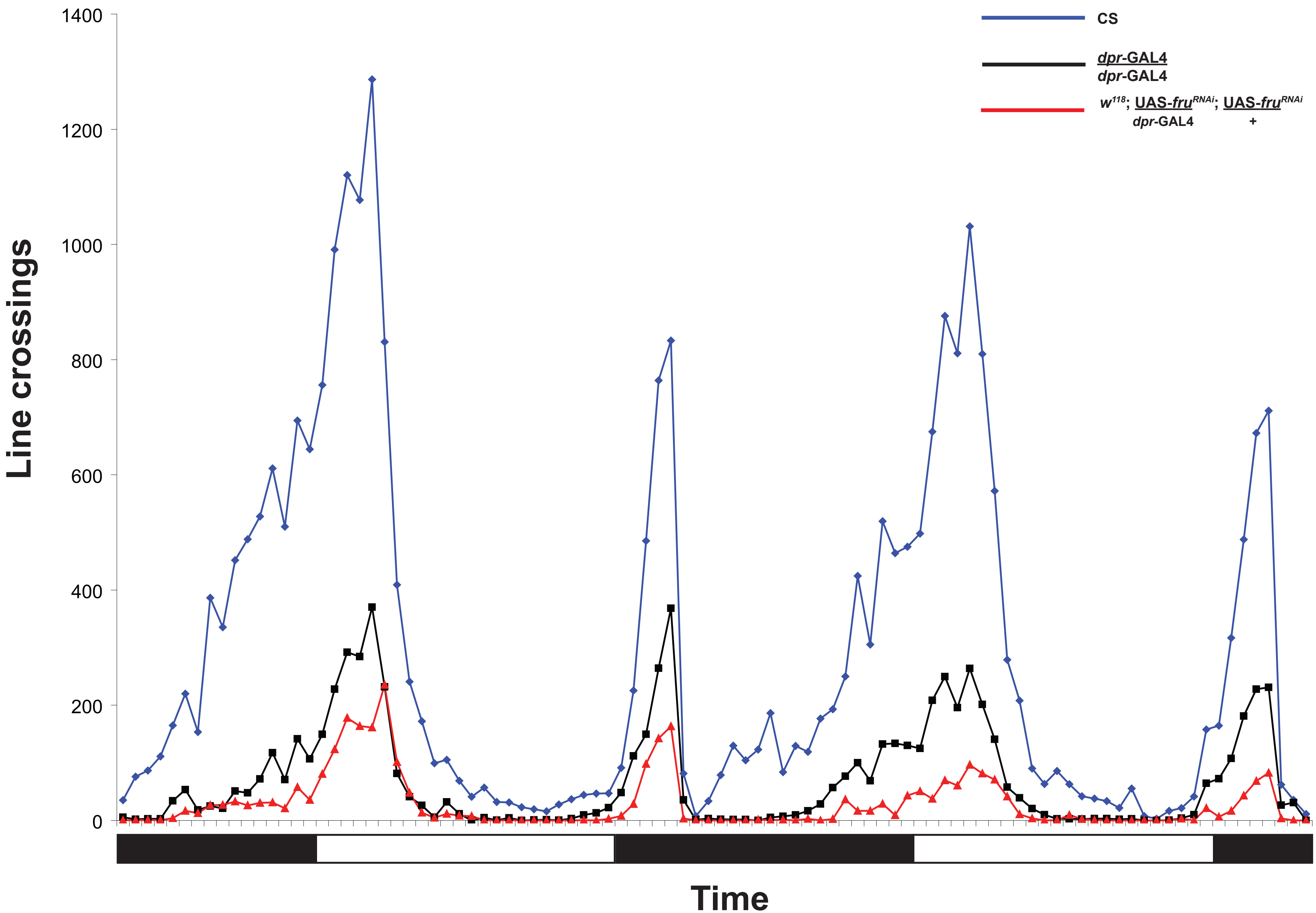

Supplement: Supplementary file 2 [file pgen.0030216.sg002.pdf]
